# The Neural Correlates of Body Image Processing in Anorexia Nervosa and Bulimia Nervosa: An Activation Likelihood Estimation Meta-Analysis of fMRI Studies

**DOI:** 10.3390/ijerph22010055

**Published:** 2025-01-01

**Authors:** Lara Norrlin, Oliver Baumann

**Affiliations:** School of Psychology, Bond University, Gold Coast, QLD 4226, Australia; lara.norrlin@student.bond.edu.au

**Keywords:** fMRI, anorexia nervosa, bulimia nervosa, eating disorder, body image

## Abstract

Body image concerns are key prognostic and pathogenic factors of anorexia nervosa (AN) and bulimia nervosa (BN). This study aimed to investigate the neural mechanisms underlying body image perception across its two domains of estimation and satisfaction in anorexia and bulimia patients and healthy controls (HC). Systematic searches were conducted across eight databases, including PubMed; Cochrane Library; Ovid; Google Scholar; Sage Journals; Scopus; PsycInfo; and ScienceDirect, from database inception until the 23rd of April 2023. The sample pertained to 14 functional magnetic resonance imaging (fMRI) studies and 556 participants, with tasks primarily including image and silhouette-based body estimation and satisfaction paradigms. ALE meta-analysis was conducted to investigate significant clusters of activation foci across the different studies. Shared activations were observed between HC, AN, and BN patients in cortical regions related to object manipulation and recognition, visuospatial awareness, and memory and negative affect regulation. Differential activation in interoceptive and higher-order cognitive or affective control regions likely hold the key to pathological body distortion. This study outlined commonalities and differences in the correlates driving healthy body mapping and eating disorder pathology. Our findings provide pertinent implications for future research, current clinical interventions, and therapeutic outcomes.

## 1. Introduction

Eating disorders are severe psychiatric conditions that impair psychosocial functioning, general health, and quality of life [1,2]. Anorexia nervosa (AN) and bulimia nervosa (BN) present during sensitive developmental periods, causing high mortality (AN = 5.1; BN = 1.7 deaths per 1000), major public health issues, and socioeconomic burdens [2,3,4,5]. AN is characterized by caloric restriction, abnormally low body weight, and a fear of weight gain, while BN denotes recurring, uncontrollable binge eating episodes and compensatory behaviors like purging or laxative/diuretic misuse to minimize weight gain [6]. While heritability accounts for 50–80% of the neurobiological vulnerabilities that, when combined with psychosocial factors, cause AN and BN, etiological inconsistency remains [1]. Rising incidences of body dissatisfaction in AN and BN are associated with social media consumption as cultural ideals of beauty, muscularity, and thinness are internalized among those predisposed to perfectionism or anxiety [7,8].

Body image is multifaceted, comprising visual, cognitive, and affective components that process mental representations of body composition and self-recognition [9,10]. While phasic body dissatisfaction is common in healthy individuals, the main difference between AN and BN is the persistent feeling of fatness, despite a normal or low body mass index (BMI) [11,12]. In AN and BN, body image dissatisfaction and perceptual incongruence concern self-objectification and the subsequent trade-offs between perceived–actual and perceived–ideal schematic comparators [9,13].

The activation of visuospatial, interoceptive, cognitive, and emotional processing centers occurs during body evaluation; thus, the insula, cingulate, temporoparietal/temporooccipital networks and fusiform gyrus are established in HCs, AN, and BN [14,15,16]. The fronto-striatal circuitry, medial prefrontal cortex, and limbic/paralimbic circuitry have been implicated in emotional appraisals of body imagery [16,17,18,19], while the precuneus and cingulate support metacognitive processing and the reinforcement of behavioral associations when evaluating body shapes [1].

Functional magnetic resonance imaging (fMRI) is a minimally invasive neuroimaging technique that has altered the landscape of eating disorder research on pathophysiology, maintenance, and treatment protocols [16]. fMRI studies denote small samples that negatively impair generalization and replication, increasing type one and two errors [20,21,22]. In body image research, the direction of disorder-specific activation remains inconsistent depending on the employed methodology, sample characteristics, and neuroimaging technique [23]. Addressing such limitations using coordinate-based meta-analyses is advantageous as correlate significance is established by pooling foci across multiple studies, improving statistical power whilst specifying key patterns of activation [24].

Establishing the mechanisms that maintain body image perception is of clinical significance, particularly following COVID-19’s significant increase in eating disorder presentation (36%) and hospitalization (48%) [25,26,27]. This ALE meta-analysis aimed to comprehensively identify and compare the neural correlates associated with body image estimation and satisfaction in AN, BN, and HC.

We hypothesized the following:

(1) AN will display greater hyperactivation in regions associated with self-referential and introspective processing, affective memory formation, and visuospatial integration when estimating body shapes.
(2) BN will convey greater hyperactivity in regions associated with attention, risk–reward processing, mentalization, memory formation, and object manipulation when estimating body shapes.
(3) HCs will display greater hyperactivation in regions responsible for object recognition, semantic memory, visual integration, and the evaluation of risk–reward when evaluating body estimation and satisfaction stimuli.
(4) AN will convey hyperactivation in regions responsible for visuospatial attention, working memory, affective regulation, and sensory integration in satisfaction paradigms.
(5) BN will display hyperactivation in regions responsible for visuo-cognitive and sensory integration, affective regulation, learning, and reward evaluation when establishing body satisfaction.

## 2. Materials and Methods

### 2.1. Literature Search, Selection, and Extraction Protocols

**Figure 1:**
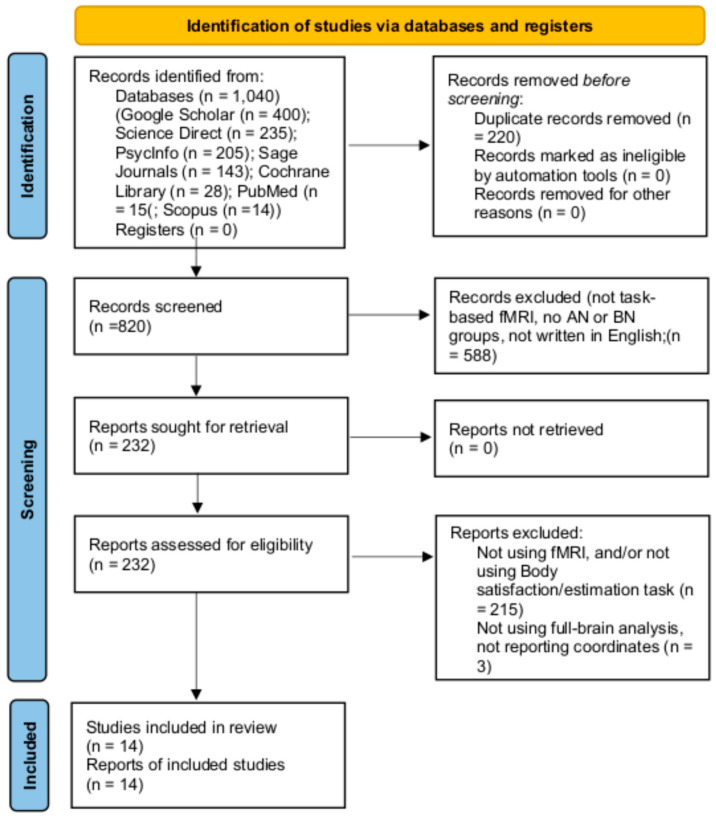
PRISMA flowchart of article search and selection procedures.

This study adhered to the Preferred Reporting Items for Systematic Reviews and Meta-Analysis (PRISMA) guidelines. We followed the PRISMA checklist to ensure transparency and completeness in the reporting of our systematic review, including specifying eligibility criteria, conducting a comprehensive literature search, screening and selecting studies, and systematically extracting and synthesizing data. The PRISMA flowchart illustrates the study selection process in detail (see Figure 1). The literature searches were performed by the 23 April 2023. The systematic review was not registered in a publicly accessible database. Articles were identified through online databases, including PubMed, Cochrane Library, Ovid, Google Scholar, Sage Journals, Scopus, PsycInfo, and ScienceDirect using the search terms: (“fMRI” OR “functional magnetic resonance imaging”) AND (“Anorexia Nervosa” OR “Bulimia Nervosa” OR “Eating Disorder”) AND (“body image perception” OR “body processing” OR “body dissatisfaction” OR “body estimation” OR “body comparison”). No year limits were enforced.

After duplicate screening, 820 articles remained. Two reviewers assessed the subsequent titles and abstracts for the following criteria: (a) written in English; (b) AN, BN, and HC groups; (c) task-based fMRI; and the following keywords: “body [image] processing/perception”, “size, shape, or weight estimation”, “body comparison”, or “body satisfaction/dissatisfaction”. Of the remaining 232 articles, one reviewer conducted a full-text review for these additional characteristics: (d) group-based comparison between AN and HC, or BN and HC; (e) image, silhouette, or priming-based body estimation and satisfaction paradigms; and (f) coordinate availability in Talairach or MNI space, producing the final sample of 14 studies (see Table 1).

### 2.2. Activation Likelihood Estimation

Activation likelihood estimation (ALE) algorithms are multivariate, coordinate-based meta-analytic strategies that determine significance by measuring the convergence of cognitive foci across fMRI studies [1,41]. Consequently, modeled activation maps are obtained by plotting each study’s foci coordinates (x, y, z) along 3D Gaussian probability distributions [41]. By converging the union calculations of activation probabilities across each voxel rather than individual cortical points, activation spreading determines whether a specified statistical threshold is met by enabling proximal activations to inflate or deflate foci significance [42,43]. To minimize bias from within- and between-group effects, GingerALE [44] implements an uncertainty of random effects model and full-width half-maximum calculations to smooth data over nearby voxels on the probability plot, which is controlled by size—where larger samples portray stronger weightings [41].

To establish reliability, the software’s ALE calculations are compared against a null distribution containing ALE scores from randomly devised permutations to produce a statistical map of *p*-values [45]. To consider the potential of multiple comparisons within the same voxel, cluster-level familywise error thresholds are employed to monitor the maximum ALE value of the distribution. For this ALE analysis, GingerALE, version 3.0.2 [44], was employed using a cluster-level familywise error threshold of 0.05, at *p* ≤ 0.05, with 1000 permutations as it represents an apt compromise for sensitivity and specificity, minimizing the risk of non-significant convergence [42]. The Lancaster transform (icbm2tal algorithm), as employed by GingerALE, was utilized to transform the Talairach coordinates into MNI space [46]. Mango, version 4.0, was utilized to visualize the significant ALE activations [47].

### 2.3. Statistical Contrasts

Due to limited hypoactivation data, hyperactivations from the 14 studies were categorized based on paradigm and participant group to assess specific elements of body estimation and satisfaction. Body estimation contrasts investigated neural response to “fat” or “thin” stimuli for AN > HC and BN > HC, while body satisfaction contrasts investigated neural sensitivity towards “own” versus “other” body comparisons for AN > HC and BN > HC. HC within-group hyperactivations were also investigated.

## 3. Results

### 3.1. Body Estimation

Significant between-group hyperactivations to “fat” body stimuli conveyed one main cluster in the right temporal and limbic lobules for AN. Regions included the fusiform gyrus, culmen, inferior temporal gyrus, and limbic lobule. Three main hyperactivation clusters, primarily in the left occipitoparietal and frontal lobules, were significant for BN. Regions included the precentral gyrus, middle and inferior frontal gyrus, insula, angular gyrus, and superior parietal lobule. Two main hyperactivation clusters, primarily in the right occipitotemporal and parietal lobule, were noted for within-group HC comparisons. Regions included the fusiform gyrus, supramarginal gyrus, superior parietal lobule, middle temporal gyrus, and superior temporal gyrus.

**Figure 2:**
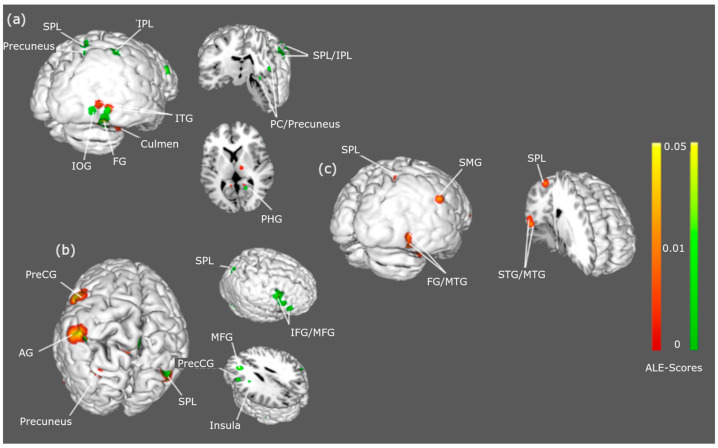
Hyperactivations to body estimation stimuli: Brain regions showing hyperactivations in AN (panel **a**) and BN (panel **b**) when viewing fat (red) and thin (green) body stimuli compared with HCs (panel **c**). Single dataset contrasts were conducted with a cluster-level familywise error of *p* < 0.05. Abbreviations: SPL = superior parietal lobule; IPL = inferior parietal lobule; IOG = inferior occipital gyrus; FG = fusiform gyrus; ITG = inferior temporal gyrus; MTG = middle temporal gyrus; PHG = parahippocampal gyrus; PC = posterior cingulate; PreCG = precentral gyrus; AG = angular gyrus; MFG = middle frontal gyrus; IFG = inferior frontal gyrus; SMG = supramarginal gyrus.

Significant hyperactivations to “thin” body stimuli were observed for AN > HC and BN > HC. For AN, two main clusters, primarily in the right temporoparietal lobule, corresponded to activation in the inferior and superior parietal lobules, precuneus, fusiform gyrus, posterior cingulate, parahippocampal gyrus, and inferior occipital gyrus. For BN, four main clusters, primarily in the right frontoparietal and temporal lobule, corresponded with activation in the precentral gyrus, middle and inferior frontal gyrus, insula, angular gyrus, and superior parietal lobule. Peak activation coordinates for each cluster, and the anatomical labels, are displayed in Table 2, while significant BOLD activations can be viewed in Figure 2.

### 3.2. Body Satisfaction

**Figure 3:**
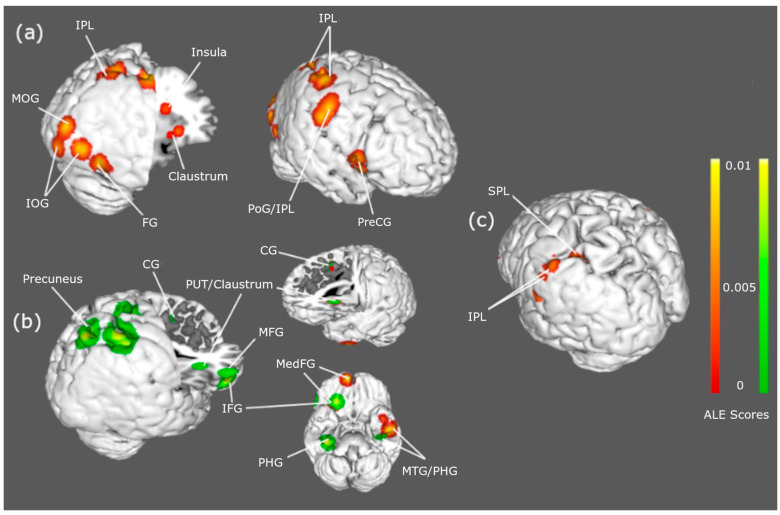
Hyperactivations to body satisfaction stimuli: Brain regions showing hyperactivations in BN (panel **b**) and AN (panel **c**) when viewing their own (red) and other (green) body stimuli compared with HCs (panel **a**). Single dataset contrasts were conducted with a cluster-level familywise error of *p* < 0.05. Abbreviations: CG = cingulate gyrus; PUT = putamen; IFG = inferior frontal gyrus; MFG = middle frontal gyrus; MedFG = medial frontal gyrus; MTG = middle temporal gyrus; PHG = parahippocampal gyrus; IPL = inferior parietal lobe; SPL = superior parietal lobe; PreCG = precentral gyrus; PoG = postcentral gyrus; MOG = middle occipital gyrus; IOG = inferior occipital gyrus; FG = fusiform gyrus.

Significant between-group hyperactivations to ‘own’ body stimuli conveyed one main cluster in the left superior and inferior parietal lobule for AN. Five main hyperactivation clusters, primarily in the right frontotemporal lobule and midbrain, were significant for BN. Regions included the middle and medial frontal gyrus, claustrum, middle temporal gyrus, putamen, and cingulate gyrus. Three main hyperactivation clusters, primarily in the right occipitoparietal and temporal lobule, were noted for within-group HC comparisons. Regions included the inferior parietal lobule, precentral and postcentral gyrus, insula, claustrum, fusiform gyrus, and the middle and inferior occipital gyrus. Significant between-group hyperactivations to “other” body stimuli were only observed for BN > HC. Six main clusters, primarily in the right frontoparietal lobule and midbrain, corresponded to activation in the precuneus, inferior frontal gyrus, claustrum, putamen, cingulate gyrus, and parahippocampal gyrus. Peak activation coordinates for each cluster and the anatomical labels are displayed in Table 3, while significant BOLD activations can be viewed in Figure 3.

## 4. Discussion

This is the first meta-analysis undertaking a coordinate-based approach to body image by investigating the neural correlates of body satisfaction and estimation in AN, BN, and HCs. Significant regions for body satisfaction included the left parietal lobe for AN; right and left frontotemporal, parietal, and midbrain regions for BN; and right occipitotemporal and parietal lobe for HCs. Significant regions for body estimation included the right temporoparietal lobe for AN; right and left occipitotemporal and frontoparietal regions for BN; and the right occipitotemporal and parietal lobe for HCs. These findings supported four hypotheses (1; 2; 3; 5), considering body estimation would be associated with regions sensitive to self-referential processing, object manipulation, visuospatial integration, and affective regulation, while body satisfaction would reflect hyperactivations among regions associated with visuospatial attention, risk–reward evaluation, and cognitive processing. One hypothesis was rejected (4), as body satisfaction in AN was not implicated with regions controlling affect regulation or visuospatial attention.

The major findings indicated activation in the fusiform gyrus, precuneus, superior/inferior parietal lobule, and inferior/superior temporal lobule were shared between AN, BN, and HCs, implicating regions in whole-body image processing, regardless of disease state. Combined with differential activation of the insula, cingulate, and frontal lobes, support arises for multisensory integration as interoceptive awareness and metacognitive evaluation appears necessary to incorporate the relevant somatosensory and visual estimates of shape-related cues [48,49,50]. Such activation patterns support that perceived-over-ideal body comparisons rely upon psychosocial adaptation to body shapes; thus, prolonged focus and affective reaction to goal-based weight management behaviors in AN and BN encapsulate two distinct cognitive processes: neural adaptation to visual aftereffects and allocentric encoding [18,51,52].

Both AN and BN employed the inferior and superior parietal lobule, which encode bodily representations and visuospatial information [53]. Notably, associations between body dysmorphia and patients with right parietal damage generate comparative levels of unilateral neglect and bodily misattribution, accounting for the dissatisfaction experienced when viewing unpleasant stimuli [53,54]. Activation of the default mode network was apparent in AN and BN during body estimation. As the precuneus and cingulate integrate semantic bodily representations and autobiographical experiences of body ownership, patients cannot avoid intrapersonal preoccupations with weight concerns—promoting attention redirection toward disorder-related stimuli [55,56]. This supports self-referential processing frameworks, which posit such mechanisms may exacerbate attentional biases towards disorder-relevant stimuli and reinforce maladaptive body image distortions [48]. Understanding these patterns suggests the importance of therapies targeting these mechanisms, such as mindfulness-based approaches that enhance interoceptive awareness and redirect attention away from maladaptive preoccupations.

During estimation tasks, AN and HCs activated the fusiform gyrus, which monitors object recognition and affective regulation of fear or surprise when viewing distorted bodily representations [36,57]. Direct lateralization with the middle occipital gyrus was observed, implicating finer-grained perceptual analyses that likely facilitated non-distortion of body schemata and mediation of adaptive shock-based responses to unexpected stimuli in HCs [15,58]. In AN, impaired somatosensory processing and preference for allocentric memorization likely prolong attention toward distressing stimuli, supporting body schemata rearrangement to meet patient ideals [52].

Affective integration was established by higher-level emotional control regions, including the culmen for AN and the precuneus and insula for BN. Cognitive control and attentional awareness were recruited by the posterior cingulate in AN, while BN engaged the angular gyrus and frontal gyri. Our findings underscore distinct neural activation patterns between anorexia nervosa (AN) and bulimia nervosa (BN). AN was characterized by heightened activation in regions such as the precuneus and parahippocampal gyrus, supporting its association with introspective and visuospatial processing [13,59,60]. These findings suggest that therapeutic approaches like mirror exposure therapy or visuospatial retraining could help recalibrate distorted body perceptions. Additionally, mindfulness-based interventions that focus on enhancing interoceptive awareness may reduce cognitive preoccupation with body image by promoting present-moment attention to bodily sensations. Such interventions may alleviate the introspective and visuospatial biases observed in AN.

In contrast, BN showed significant activation in regions like the insula and angular gyrus, reflecting the disorder’s emphasis on affect regulation and social comparison during body image tasks [1,4,34]. The observed activation of the insula and angular gyrus highlights the importance of therapies that target emotion regulation and social cognition. For example, dialectical behavior therapy (DBT) could be used to address difficulties in regulating emotions triggered by body comparison, while cognitive interventions aimed at reducing the internalization of societal ideals may help mitigate the negative impact of maladaptive social comparisons on body dissatisfaction. These differences likely reflect the unique cognitive and affective mechanisms underlying the two disorders.

The culmen sits in the anterior cerebellar vermis with extensive connections to the parahippocampal gyrus and cingulate, which were coactivations in AN [61]. This unexpected activation may reflect distinct aspects of psychopathology, as conveyed in anxiety and mood disorders [61,62,63]. Insula activity in BN during body comparison is consistent with previous findings, emphasizing attentional redirection to one’s own bodily flaws after observing other bodily contours [1]. This likely reflects developed responses to reward signals associated with weight reduction or idealized constructs acquired from familial and peer socialization [1,14,64]. Activation of the insula during body comparison tasks aligns with social comparison and affective appraisal theories, highlighting attentional redirection towards perceived bodily flaws and the influence of societal ideals [7,40]. These mechanisms likely contribute to the reinforcement of maladaptive behaviors, such as body-checking and weight-management strategies, particularly in individuals with bulimia nervosa.

By delineating disorder-specific neural activation patterns, this study underscores the need for tailored therapeutic interventions. Integrative approaches, such as integrative cognitive–affective therapy (ICAT), may address the unique cognitive and affective challenges in AN and BN while leveraging shared neural pathways to improve body image and reduce maladaptive behaviors. Incorporating insights from this research into clinical practice could enhance the efficacy of existing treatments and foster the development of new, targeted strategies.

The parahippocampus controls personal perspectives when viewing body images and complex emotional processing, particularly of food-based rewards [65]. In the body image context, activation across both eating disorder cohorts may highlight a potential root cause of pathology. When combined with the parieto-temporal and frontal activations that drive the multisensory direction of attention toward the relevant areas of dislike on one’s own body, it is conceivable that multifaceted bottom-up processes of analysis occur when engaging in negative social comparisons [59,66]. These findings underscore the importance of addressing both perceptual and emotional distortions in therapeutic interventions, particularly in relation to maladaptive reward processing and social comparison behaviors. For instance, cognitive–behavioral interventions could aim to restructure these bottom-up processes to reduce habitual negative reinforcement mechanisms, such as purging or body checking behaviors.

For example, to dampen anxiety when viewing undesirable bodies, introspective responses likely trigger various weight-management (purging and starvation) or body-checking behaviors (pinching) via reinforcement, which become habitual coping mechanisms, defining pathophysiology [40]. This interplay between cognitive, emotional, and behavioral responses highlights the necessity for comprehensive treatment strategies that integrate cognitive restructuring with techniques targeting emotional regulation and habitual behaviors.

Activation of the middle and inferior frontal gyri, which control working memory and fear recognition [58,67] in BN and HCs, highlights potential cognitive deficits driving poor decision-making in AN. As the frontal regions are implicated in the habit formation of maladaptive food behaviors, hypoactivation may reflect a need for top-down control when manipulating spatial representations of body imagery, giving rise to complex dilemmas between “wanting” their body to look a certain way and “liking” the way their body looks/feels based on social norms, demands, and self-worth contingencies [31,37,68]. These findings suggest that addressing working memory deficits and decision-making challenges through interventions like executive function training could be particularly beneficial for AN. Furthermore, therapies that incorporate motivational interviewing might help patients reconcile conflicts between internalized ideals and personal satisfaction.

Given the similarity in activation between AN and BN, this questions the weighting that neurobiological and personality-related factors, like perfectionism and persistence, play in influencing the severity of body distortion and pathology [19,69]. Analyses of HC data consolidated neurotypical body mapping, evidencing the absence of multisensory impairments and processing biases observed in AN and BN. Consequently, phasic body dissatisfaction likely represents temporary homuncular distortions of length and volume perception before cognitive and affective disengagement to avoid distress [12]. The findings in HCs suggest that intact multisensory integration and adaptive cognitive disengagement serve as protective factors against body dissatisfaction. These insights could inform preventative strategies for at-risk populations, emphasizing interventions that strengthen these protective mechanisms.

By highlighting the similar and distinguishing features between HCs, AN, and BN in an exploratory, quantitative meta-analytic approach, this study provided a comprehensive summary of the neural mechanisms driving body image distortion and dissatisfaction. This delineates clinical implications for the modulation of future and restructuring of current interventions, like cognitive–behavioral body image therapy, to enhance therapeutic outcomes [10,27,60]. Further interdisciplinary research should explore how these neural mechanisms interact with environmental and psychosocial factors, such as cultural pressures and interpersonal relationships, to develop more holistic approaches to treatment.

The heterogeneity of studies in meta-analyses is undesirable; to produce valid conclusions, consistency between the selected studies is necessary [70]. Stringent inclusion and exclusion criteria across age, gender, and group BMI were employed; however, disease duration was difficult to control, which can limit generalizability. Given the similarity between average disease duration (Median AN = 5.9 years; Median BN = 6.4 years), bias is unlikely. Including word-based body image descriptions, video stimuli, and body-checking tasks might introduce differential effects, as these stimuli can evoke varying levels of emotional and cognitive engagement [37]. Regardless, pooling data across several categories and studies did not constrain analyses, facilitating the use of stringent statistical thresholding, thus indicating a sufficiently large evidence base to generate confidence in result reliability.

We highlighted possible neurobiological and personality mechanisms accounting for some differences in body image perception among AN and BN. While studies exist on both topics more broadly, particularly concerning appetitive cues [71], very few have been combined with task-based fMRI studies on body image. Splitting AN by its restricting and purging subtypes may provide additional information on disorder manifestation and cross-over as the AN binge–purge type aligns with typical BN symptomology [72,73]. Future interdisciplinary research should also consider integrating neuroimaging with nutritional, behavioral, and psychosocial factors to develop a more comprehensive understanding of eating disorder pathology.

Dietary patterns and nutrient intake have been linked to mental health outcomes, though their role in eating disorders remains unclear. For instance, diets characterized by high intakes of processed foods, added sugars, and saturated fats have been associated with increased risks of depression and anxiety [74]. In contrast, adherence to healthier dietary patterns, such as the Mediterranean diet, has been linked to improved mental health outcomes [75]. While these associations suggest that diet may influence factors relevant to mental health, it is important to note that no direct causal links to eating disorders have been established. Future research could explore whether dietary patterns or specific nutrient exposures interact with neural mechanisms underlying body image perception, but such investigations are beyond the scope of this study.

Our findings may have clinical implications, particularly for advancing the understanding of the neural mechanisms underlying body image disturbances in individuals with eating disorders. By identifying patterns of neural activation associated with body image estimation and satisfaction, this study contributes to the growing body of knowledge that can inform targeted therapeutic approaches. For example, insights into the distinct neural mechanisms of AN and BN could guide the development of tailored interventions, such as visuospatial retraining for AN or emotion regulation therapies for BN. These approaches could be further enhanced by incorporating evidence-based strategies that address shared pathways, such as cognitive–affective integration therapies, to improve treatment outcomes across both disorders.

Although this study does not directly examine nutritional factors, future interdisciplinary research could explore the integration of neurobiological findings with nutritional and behavioral interventions. By combining neuroscientific insights with broader behavioral and environmental data, personalized treatment strategies could be developed to complement existing psychological and pharmacological therapies, offering a more holistic approach to addressing eating disorders.

## 5. Conclusions

This review’s findings implicated a range of underlying mechanisms in neurotypical and pathological body image processing between AN, BN, and HCs, highlighting how multisensory integration deficits, impaired introspective awareness, and metacognitive evaluation contribute to distorted body estimation and satisfaction. These mechanisms exacerbate affective responses and attention to disorder-related cues. These results provide clinical and empirical implications to facilitate understanding of disorder trajectory, prognosis, and the modulation of body image therapies to reduce relapse and enhance therapeutic outcomes.

## Figures and Tables

**Table 1:** Data Extraction and Summary of Studies Included in the Meta-Analysis.

Study	Participants	AN Subtype	Duration of Illness	BMI	Age	fMRI Field Strength	Task
Castellini et al. (2013) [28]	18 with AN 19 HC	All AN-R	5.6 years	16.1 20.8	24.9 26.8	1.5 Tesla	Viewing photographs of distorted self-body images vs. photographs of houses.
Friederich et al. (2010) [29]	17 with AN 18 HC		7.2 years	15.6 21.9	24.9 23.3	1.5 Tesla	Viewing images of thin models vs. interior design images before rating anxiety level.
Horndasch et al. (2020) [15]	15 adolescents with AN 18 adolescent HCs 19 adults with AN 17 adult HCs		1.4 years for adolescents 6.1 years for adults	5.33 * 56.33 * 16.06 21.40	15.72 16.45 26.27 27.03	3 Tesla	Viewing images of emaciated and obese bodies in front vs. rear perspectives before rating satisfaction.
Miyake et al. (2010a) [30]	22 with AN 11 with BN 11 HC	11 AN-R, 11 AN-BP	4.1 years 7.9 years 6.4 years	15.3 15.9 20.2 19.1	22.2 28.3 24.5 26.5	1.5 Tesla	Viewing photographs of distorted self vs. other bodies and rating dislike.
Miyake et al. (2010b) [31]	24 with AN 12 with BN 12 HC	12 AN-R, 12 AN-BP	5.3 years 6.4 years 5.1 years	15.2 15.5 21.2 18.8	27 27.2 25 25.4	1.5 Tesla	Viewing negative body image words vs. neutral words.
Mohr et al. (2010) [32]	16 with AN 16 HC			15.9 21.7	24.1 25.6	3 Tesla	Viewing distorted photographs of self vs. other bodies before rating similarity.
Mohr et al. (2011) [33]	15 with BN 16 HC			22.0 21.8	24.8 25.5	3 Tesla	Viewing distorted photographs of self vs. other bodies before rating similarity.
Sachdev et al. (2008) [34]	10 with AN 10 HC	5 AN-R, 5 AN-BP	Between 1 and 4 years	16.40 22.17	18.8 22.6	3 Tesla	Viewing photographs of self vs. other bodies from different angles.
Spangler and Allen (2012) [35]	12 with BN 12 HC			19.3–27.8 18.5–26.9	18–38 18–30	1.5 Tesla	Viewing digital silhouettes of thin vs. fat bodies.
Suda et al. (2013) [36]	20 with AN 15 HC		5.4 years	15.7 21.9	27.0 26.0	1.5 Tesla	Viewing photographs of body checking tasks vs. active tasks.
Uher et al. (2005) [37]	13 with AN 9 with BN 18 HC	7 AN-R, 6 AN-BP	11.8 years 14.2 years	16.2 22.6	25.4 29.6 26.6	1.5 Tesla	Viewing line drawings of underweight, normal, and overweight bodies vs. house drawings.
Van Den Eynde et al. (2013) [38]	21 with BN 23 HC		Primarily between 5 and 10 years	23.4 21.3	28.0 27.3	1.5 Tesla	Viewing thin body images vs. interior design images.
Via et al. (2018) [39]	21 with AN 21 HC	All AN-R	7.1 years	16.9 20.9	28.4 28.2	1.5 Tesla	Viewing video clips of self vs. other bodies.
Vocks et al. (2010) [40]	13 with AN 15 with BN 27 HC	All AN-BP	7.2 years 11.8 years	15.78 21.34 22.06	29.08 28.40 26.74	1.5 Tesla	Viewing photographs of self vs. other bodies from different angles.

Note. * BMI of adolescents was reported in age percentiles. Abbreviations: AN-BP = anorexia nervosa binge–purge subtype; AN-R = anorexia nervosa restrictive subtype.

**Table 2:** Summary of Significant ALE Findings for Body Estimation.

Group	Cluster Number	MNI Coordinates			*Z*	Region	BA	Cluster Size
		*X*	*Y*	*Z*				Vol/mm^3^
Anorexia (Fat)								
	1	42	−62	−12	4.853	R. Fusiform Gyrus (Temporal)	37	14,216
		48	−50	−20	3.534	R. Culmen	-	
		50	−70	2	3.386	R. Inferior Temporal Gyrus	37	
		26	−54	−4	3.315	R. Parahippocampal Gyrus	19	
Anorexia (Thin)								
	1	50	−48	46	3.657	R. Inferior Parietal Lobule	40	18,752
		38	−42	46	3.601	R. Inferior Parietal Lobule	40	
		30	−52	50	3.584	R. Superior Parietal Lobule	7	
		28	−64	58	3.508	R. Superior Parietal Lobule	7	
		48	−36	48	3.499	R. Inferior Parietal Lobule	40	
		32	−68	32	3.448	R. Precuneus	7	
		28	−62	48	3.186	R. Superior Parietal Lobule	7	
	2	42	−60	−14	5.635	R. Fusiform Gyrus (Temporal)	37	18,576
		26	−54	−2	4.012	R. Parahippocampal Gyrus	19	
		46	−48	−16	3.856	R. Fusiform Gyrus (Temporal)	37	
		16	−52	8	3.568	R. Posterior Cingulate	29	
		48	−76	−4	3.487	R. Inferior Occipital Gyrus	19	
Healthy (Fat)								
	1	44	−48	−16	4.031	R. Fusiform Gyrus (Temporal)	37	13,720
		46	−68	−6	3.861	R. Fusiform Gyrus (Occipital)	19	
		46	−60	−6	3.657	R. Middle Temporal Gyrus	37	
		44	−64	−12	3.650	R. Fusiform Gyrus (Temporal)	37	
	2	60	−44	30	3.828	R. Supramarginal Gyrus	40	11,496
		30	−62	48	3.532	R. Superior Parietal Lobule	7	
		48	−52	34	3.202	R. Superior Temporal Gyrus	39	
Bulimia (Fat)								
	1	−12	−54	50	3.699	L. Precuneus	7	10,872
		−28	−62	42	3.273	L. Precuneus	7	
	2	34	−54	40	3.879	R. Angular Gyrus	39	10,048
		30	−62	48	3.347	R. Superior Parietal Lobule	7	
	3	−52	2	38	4.071	L. Precentral Gyrus	6	8640
		−48	4	32	3.687	L. Precentral Gyrus	6	
Bulimia (Thin)								
	1	50	8	36	4.003	R. Precentral Gyrus	6	12,520
		50	26	16	3.351	R. Middle Frontal Gyrus	46	
		50	14	26	3.092	R. Inferior Frontal Gyrus	9	
	2	−36	−10	20	3.539	L. Insula	13	9144
		−46	4	30	3.064	L. Precentral Gyrus	6	
	3	36	−56	38	3.354	R. Angular Gyrus	39	8912
		30	−62	48	3.088	R. Superior Parietal Lobule	7	
	4	−28	18	42	3.813	L. Middle Frontal gyrus	8	8392
		−26	26	28	3.404	L. Middle Frontal Gyrus	9	

Note. Anatomical locations, spatial coordinates, and ALE scores of significant activations (with a cluster-level familywise error of *p* < 0.05). L = left hemisphere; R = right hemisphere. BA = Brodmann area.

**Table 3:** Summary of Significant ALE Findings for Body Satisfaction.

Group	Cluster Number	MNI Coordinates			ALE Score	*p*	*Z*	Region	BA	Cluster Size
		*X*	*Y*	*Z*						Vol/mm^3^
Anorexia (Own)										
	1	−38	−50	48	0.0117	5.68 × 10^−6^	4.390	L. Inferior Parietal Lobule	40	14,864
		−52	−42	50	0.0087	5.71 × 10^−5^	3.858	L. Inferior Parietal Lobule	40	
		−30	−54	52	0.0086	5.86 × 10^−5^	3.852	L. Superior Parietal Lobule	7	
		−54	−38	42	0.0085	1.67 × 10^−4^	3.588	L. Inferior Parietal Lobule	40	
		−60	−34	26	0.0085	1.67 × 10^−4^	3.588	L. Inferior Parietal Lobule	40	
		−48	−46	60	0.0075	2.90 × 10^−4^	3.441	L. Inferior Parietal Lobule	40	
		−36	−58	64	0.0074	8.88 × 10^−4^	3.125	L. Superior Parietal Lobule	7	
Healthy (Own)										
	1	62	−22	26	0.0088	5.97 × 10^−5^	3.847	R. Inferior Parietal Lobule	40	20,064
		56	−34	50	0.0084	1.37 × 10^−4^	3.639	R. Inferior Parietal Lobule	40	
		39	−52.5	47	5.50 × 10^−4^	0.049	1.658	R. Inferior Parietal Lobule	40	
		62	−20	36	0.0069	7.72 × 10^−4^	3.166	R. Postcentral Gyrus	2	
	2	42	10	−8	0.0084	1.37 × 10^−4^	3.639	R. Insula	14	13,664
		33	24	−3	0.0023	0.017	2.111	R. Claustrum	-	
		56	10	8	0.0066	0.001	3.057	R. Precentral Gyrus	44	
	3	36	−90	12	0.0084	7.13 × 10^−5^	3.804	R. Middle Occipital Gyrus	19	12,456
		46	−80	0	0.0084	1.37 × 10^−4^	3.639	R. Inferior Occipital Gyrus	19	
		45	−63	−9	0.0064	0.002	2.888	R. Fusiform Gyrus (Temporal)	37	
		36	−96	−2	0.0066	0.001	3.057	R. Inferior Occipital Gyrus	18	
BN (Own)										
	1	24	26	−20	0.0077	2.48 × 10^−5^	4.057	R. Middle Frontal Gyrus	11	26,396
		30	20	2	0.0075	3.41 × 10^−5^	3.982	R. Claustrum	-	
	2	−44	−0.7	−40	6.63 × 10^−4^	0.011	2.274	L. Middle Temporal Gyrus	21	20,120
	3	−28	11	3	0.0073	6.94 × 10^−5^	3.810	L. Lentiform Nucleus/putamen	-	15,648
	4	11	10	43	0.0073	6.94 × 10^−5^	3.810	R. Cingulate Gyrus	24	15,648
	5	8	58	−16	0.0077	2.48 × 10^−5^	4.057	R. Medial Frontal Gyrus	10	12,112
BN (Other)										
	1	33	−48	56.5	3.17 × 10^−4^	0.025	1.963	R. Precuneus	7	29,832
	2	54	32	4	0.0077	4.03 × 10^−5^	3.943	R. Inferior Frontal Gyrus	45	
		30	20	2	0.0075	5.00 × 10^−5^	3.891	R. Claustrum	-	
	3	−28	11	3	0.0073	8.55 × 10^−5^	3.758	R. Lentiform Nucleus/putamen	-	9824
	4	11	10	43	0.0073	8.55 × 10^−5^	3.758	R. Cingulate Gyrus	24	9824
	5	−34	−12	−22	0.0077	4.03 × 10^−5^	3.943	L. Parahippocampal Gyrus	-	9704
	6	34	−24	−28	0.0077	4.03 × 10^−5^	3.943	R. Parahippocampal Gyrus	36	7560

Note. Anatomical locations, spatial coordinates, and ALE scores of significant activations (with a cluster-level family-wise error of *p* < 0.05). L = Left hemisphere; R = Right hemisphere. BA = Brodmann area.

## Data Availability

No new data were created or analyzed in this study.

## References

[1] McAdams C.J., Krawczyk D.C. (2014). Who am I? How do I look? Neural differences in self-identity in anorexia nervosa. Soc. Cogn. Affect. Neurosci..

[2] Zhang Z., Robinson L.M., Jia T., Quinlan E.B., Tay N., Chu C., Barker E.D., Banaschewski T., Barker G.J., Bokde A.L. (2021). Development of disordered eating behaviors and comorbid depressive symptoms in adolescence: Neural and psychopathological predictors. Biol. Psychiatry.

[3] Ágh T., Kovacs G.G., Supina D., Pawaskar M., Herman B.K., Vokó Z., Sheehan D.V. (2016). A systematic review of the health-related quality of life and economic burdens of anorexia nervosa, bulimia nervosa, and binge eating disorder. Eat. Weight. Disord.-Stud. Anorex. Bulim. Obes..

[4] Nagl M., Jacobi C., Paul M., Beesdo-Baum K., Höfler M., Lieb R., Wittchen H. (2016). Prevalence, incidence, and natural course of anorexia and bulimia nervosa among adolescents and young adults. Eur. Child. Adolesc. Psychiatry.

[5] Van Eeden A.E., Van Hoeken D., Hoek H.W. (2021). Incidence, prevalence and mortality of anorexia nervosa and bulimia nervosa. Curr. Opin. Psychiatry.

[6] American Psychiatric Association (2013). Diagnostic and Statistical Manual of Mental Disorders.

[7] Ferguson C.J., Muñoz M., Garza A., Galindo M. (2014). Concurrent and prospective analyses of peer, television, and social media influences on body dissatisfaction, eating disorder symptoms and life satisfaction in adolescent girls. J. Youth Adolesc..

[8] Van Den Berg P., Paxton S.J., Keery H., Wall M.M., Guo J., Neumark-Sztainer D. (2007). Body dissatisfaction and body comparison with media images in males and females. Body Image.

[9] Hamamoto Y., Suzuki S., Motoki K., Oba K., Kawashima R., Sugiura M. (2023). Neural mechanisms of perceptual and affective body-image disturbance during own-body and ideal-body estimation. Behav. Brain Res..

[10] Zanetti T., Santonastaso P., Sgaravatti E., Degortes D., Favaro A. (2013). Clinical and temperamental correlates of body image disturbance in eating disorders. Eur. Eat. Disord. Rev..

[11] Riva G. (2016). Neurobiology of anorexia nervosa: Serotonin dysfunctions link self-starvation with body image disturbances through an impaired body memory. Front. Human. Neurosci..

[12] Sadibolova R., Ferrè E.R., Linkenauger S.A., Longo M.R. (2019). Distortions of perceived volume and length of body parts. Cortex.

[13] Jongenelis M.I., Byrne S.M., Pettigrew S. (2014). Self-objectification, body image disturbance, and eating disorder symptoms in young Australian children. Body Image.

[14] Gaudio S., Quattrocchi C.C. (2012). Neural basis of a multidimensional model of body image distortion in anorexia nervosa. Neurosci. Biobehav. Rev..

[15] Horndasch S., Rösch J., Kratz O., Vogel A., Heinrich H., Graap H., Moll G.H., Dörfler A., Forster C. (2020). Neural mechanisms of perceptive and affective processing of body stimuli in anorexia nervosa—Are there developmental effects?. Psychiatry Res..

[16] Steward T., Menchón J.M., Jiménez-Murcia S., Soriano-Mas C., Fernández-Aranda F. (2017). Neural network alterations across eating disorders: A narrative review of fMRI studies. Curr. Neuropharmacol..

[17] Collantoni E., Alberti F., Meregalli V., Meneguzzo P., Tenconi E., Favaro A. (2021). Brain networks in eating disorders: A systematic review of graph theory studies. Eat. Weight. Disord.-Stud. Anorex. Bulim. Obes..

[18] Grace S.A., Labuschagne I., Kaplan R.A., Rossell S.L. (2017). The neurobiology of body dysmorphic disorder: A systematic review and theoretical model. Neurosci. Biobehav. Rev..

[19] Mishra A.K., Anand M., Umesh S. (2017). Neurobiology of eating disorders: An overview. Asian J. Psychiatry.

[20] Donnelly B., Touyz S., Hay P., Burton A., Russell J., Caterson I. (2018). Neuroimaging in bulimia nervosa and binge eating disorder: A systematic review. J. Eat. Disord..

[21] Fonville L., Giampietro V., Williams S.C., Simmons A., Tchanturia K. (2014). Alterations in brain structure in adults with anorexia nervosa and the impact of illness duration. Psychol. Med..

[22] Turner B.L., Paul E.J., Miller M.I., Barbey A.K. (2018). Small sample sizes reduce the replicability of task-based fMRI studies. Commun. Biol..

[23] Lavagnino L., Amianto F., D’Agata F., Huang Z., Mortara P., Abbate-Daga G., Marzola E., Spalatro A.V., Fassino S., Northoff G. (2014). Reduced resting-state functional connectivity of the somatosensory cortex predicts psychopathological symptoms in women with bulimia nervosa. Front. Behav. Neurosci..

[24] Samartsidis P., Montagna S., Nichols T.E., Johnson T.D. (2017). The coordinate-based meta-analysis of neuroimaging data. Stat. Sci. A Rev. J. Inst. Math. Stat..

[25] Chang P.G., Delgadillo J., Waller G. (2021). Early response to psychological treatment for eating disorders: A systematic review and meta-analysis. Clin. Psychol. Rev..

[26] Devoe D.J., Han A., Anderson A., Katzman D.K., Patten S.B., Soumbasis A., Flanagan J., Paslakis G., Vyver E., Marcoux G. (2022). The impact of the COVID-19 pandemic on eating disorders: A systematic review. Int. J. Eat. Disord..

[27] Von Hausswolff-Juhlin Y., Brooks S.J., Larsson M. (2015). The neurobiology of eating disorders—A clinical perspective. Acta Psychiatr. Scand..

[28] Castellini G., Polito C., Bolognesi E., D’Argenio A., Ginestroni A., Mascalchi M., Pellicanò G., Mazzoni L.N., Rotella F., Faravelli C. (2013). Looking at my body. Similarities and differences between anorexia nervosa patients and controls in body image visual processing. Eur. Psychiatry.

[29] Friederich H.C., Brooks S., Uher R., Campbell I.C., Giampietro V., Brammer M., Williams S.C., Herzog W., Treasure J. (2010). Neural correlates of body dissatisfaction in anorexia nervosa. Neuropsychologia.

[30] Miyake Y., Okamoto Y., Onoda K., Kurosaki M., Shirao N., Okamoto Y., Yamawaki S. (2010). Brain activation during the perception of distorted body images in eating disorders. Psychiatry Res. Neuroimaging.

[31] Miyake Y., Okamoto Y., Onoda K., Shirao N., Okamoto Y., Otagaki Y., Yamawaki S. (2010). Neural processing of negative word stimuli concerning body image in patients with eating disorders: An fMRI study. NeuroImage.

[32] Mohr H.M., Zimmermann J., Röder C., Lenz C., Overbeck G., Grabhorn R. (2010). Separating two components of body image in anorexia nervosa using fMRI. Psychol. Med..

[33] Mohr H.M., Röder C., Zimmermann J., Hummel D., Negele A., Grabhorn R. (2011). Body image distortions in bulimia nervosa: Investigating body size overestimation and body size satisfaction by fMRI. NeuroImage.

[34] Sachdev P.S., Mondraty N., Wen W., Gulliford K. (2008). Brains of anorexia nervosa patients process self-images differently from non-self-images: An fMRI study. Neuropsychologia.

[35] Spangler D.L., Allen M.G. (2012). An fMRI investigation of emotional processing of body shape in bulimia nervosa. Int. J. Eat. Disord..

[36] Suda M., Brooks S.J., Giampietro V., Friederich H., Uher R., Brammer M., Williams S., Campbell I.L., Treasure J. (2013). Functional neuroanatomy of body checking in people with anorexia nervosa. Int. J. Eat. Disord..

[37] Uher R., Murphy T., Friederich H., Dalgleish T., Brammer M., Giampietro V., Phillips M.L., Andrew C., Ng V., Williams S. (2005). Functional neuroanatomy of body shape perception in healthy and eating-disordered women. Biol. Psychiatry.

[38] Van Den Eynde F., Giampietro V., Simmons A., Uher R., Andrew C., Harvey P., Campbell I.L., Schmidt U. (2013). Brain responses to body image stimuli but not food are altered in women with bulimia nervosa. BMC Psychiatry.

[39] Via E., Goldberg X., Sánchez I., Forcano L., Harrison B.J., Davey C.G., Pujol J., Martínez-Zalacaín I., Fernández-Aranda F., Soriano-Mas C. (2018). Self and other body perception in anorexia nervosa: The role of posterior DMN nodes. World J. Biol. Psychiatry.

[40] Vocks S., Busch M., Grönemeyer D., Schulte D., Herpertz S., Suchan B. (2010). Neural correlates of viewing photographs of one’s own body and another woman’s body in anorexia and bulimia nervosa: An fMRI study. J. Psychiatry Neurosci..

[41] Eickhoff S.B., Bzdok D., Laird A.R., Kurth F., Fox P.T. (2012). Activation likelihood estimation meta-analysis revisited. NeuroImage.

[42] Eickhoff S.B., Nichols T.E., Laird A.R., Hoffstaedter F., Amunts K., Fox P.T., Bzdok D., Eickhoff C.R. (2016). Behavior, sensitivity, and power of activation likelihood estimation characterized by massive empirical simulation. NeuroImage.

[43] Laird A.R., Eickhoff S.B., Kurth F., Fox P.T., Uecker A.M., Calhoun V.D., Colhoun H.M., Lancaster J.L. (2009). ALE meta-analysis workflows via the BrainMap database: Progress towards a probabilistic functional brain atlas. Front. Neuroinformatics.

[44] Turkeltaub P.E., Eden G.F., Jones K., Zeffiro T.A. (2002). Meta-analysis of the functional neuroanatomy of single word reading: Method and validation. NeuroImage.

[45] Zhu Y., Hu X., Wang J., Chen J., Guo Q., Li C., Enck P. (2012). Processing of food, body, and emotional stimuli in anorexia nervosa: A systematic review and meta-analysis of functional magnetic resonance imaging studies. Eur. Eat. Disord. Rev..

[46] Laird A.R., Colhoun H.M., McMillan K.A., Tordesillas-Gutiérrez D., Moran S.T., Gonzales S.M., Ray K.L., Franklin C., Glahn D.C., Fox P.T. (2010). Comparison of the disparity between Talairach and MNI coordinates in functional neuroimaging data: Validation of the Lancaster transform. NeuroImage.

[47] Lancaster J.L., Martinez M.J. (2020). Mango (Version 4.1) [Computer Software].

[48] Badoud D.M., Tsakiris M. (2017). From the body’s viscera to the body’s image: Is there a link between interoception and body image concerns?. Neurosci. Biobehav. Rev..

[49] Fernandez-Duque D., Baird J.A., Posner M.I. (2000). Executive attention and metacognitive regulation. Conscious. Cogn..

[50] Stern E., Grimaldi S.J., Muratore A.F., Murrough J., Leibu E., Fleysher L., Goodman W.K., Burdick K.E. (2017). Neural correlates of interoception: Effects of interoceptive focus and relationship to dimensional measures of body awareness. Hum. Brain Mapp..

[51] Hummel D., Rudolf A.K., Brandi M., Untch K., Grabhorn R., Hampel H., Mohr H.M. (2012). Neural adaptation to thin and fat bodies in the fusiform body area and middle occipital gyrus: An fMRI adaptation study. Hum. Brain Mapp..

[52] Press S.A., Biehl S.C., Domes G., Svaldi J. (2022). Increased insula and amygdala activity during selective attention for negatively valenced body parts in binge eating disorder. J. Psychopathol. Clin. Sci..

[53] Case L.E., Wilson R., Ramachandran V.S. (2011). Diminished size-weight illusion in anorexia nervosa: Evidence for visuo-proprioceptive integration deficit. Exp. Brain Res..

[54] Tomasino S.J. (1996). Does right parietal cortex and vestibular dysfunction underlie body image distortion?. J. Nerv. Ment. Dis..

[55] Cavanna A.E., Trimble M.R. (2006). The precuneus: A review of its functional anatomy and behavioural correlates. Brain.

[56] Domakonda M., He X., Lee S., Cyr M., Marsh R. (2019). Increased functional connectivity between ventral attention and default mode networks in adolescents with bulimia nervosa. J. Am. Acad. Child. Adolesc. Psychiatry.

[57] Cross E.S., Mackie E.C., Wolford G.L., De CHamilton A.F. (2009). Contorted and ordinary body postures in the human brain. Exp. Brain Res..

[58] Suchan B., Bauser D.S., Busch M., Schulte D., Grönemeyer D., Herpertz S., Vocks S. (2013). Reduced connectivity between the left fusiform body area and the extrastriate body area in anorexia nervosa is associated with body image distortion. Behav. Brain Res..

[59] Fuentes C.T., Longo M.R., Haggard P. (2013). Body image distortions in healthy adults. Acta Psychol..

[60] Morgan J., Lazarova S., Schelhase M., Saeidi S. (2013). Ten session body image therapy: Efficacy of a manualised body image therapy. Eur. Eat. Disord. Rev..

[61] Amianto F., D’Agata F., Lavagnino L., Caroppo P., Abbate-Daga G., Righi D., Scarone S., Bergui M., Mortara P., Fassino S. (2013). Intrinsic connectivity networks within cerebellum and beyond in eating disorders. Cerebellum.

[62] Hagura N., Oouchida Y., Aramaki Y., Okada T., Matsumura M., Sadato N., Naito E. (2008). Visuokinesthetic perception of hand movement is mediated by cerebro-cerebellar interaction between the left cerebellum and right parietal cortex. Cereb. Cortex.

[63] Sacchetti B., Scelfo B., Strata P. (2009). Cerebellum and emotional behavior. Neuroscience.

[64] Lawler M., Nixon E. (2011). Body dissatisfaction among adolescent boys and girls: The effects of body mass, peer appearance, culture, and internalization of appearance ideals. J. Youth Adolesc..

[65] Bohon C., Stice E., Spoor S.T. (2009). Female emotional eaters show abnormalities in consummatory and anticipatory food reward: A functional magnetic resonance imaging study. Int. J. Eat. Disord..

[66] Mohr H.M., Rickmeyer C., Hummel D., Ernst M., Grabhorn R. (2016). Altered visual adaptation to body shape in eating disorders: Implications for body image distortion. Perception.

[67] Favaro A., Santonastaso P., Manara R., Bosello R., Bommarito G., Tenconi E., Di Salle F. (2012). Disruption of visuospatial and somatosensory functional connectivity in anorexia nervosa. Biol. Psychiatry.

[68] Reichel V.A., Schneider N., Grünewald B., Kienast T., Pfeiffer E., Lehmkuhl U., Korte A. (2013). “Glass fairies” and “bone children”: Adolescents and young adults with anorexia nervosa show positive reactions towards extremely emaciated body pictures measured by the startle reflex paradigm. Psychophysiology.

[69] Díaz-Marsá M., Carrasco J.L.E., Saiz J. (2000). A study of temperament and personality in anorexia and bulimia nervosa. J. Personal. Disord..

[70] Serdar C.C., Cihan M., Yücel D., Serdar M. (2021). Sample size, power, and effect size revisited: Simplified and practical approaches in pre-clinical, clinical and laboratory studies. Biochem. Med..

[71] Grimm O., Jacob M.J., Kroemer N.B., Krebs L., Vollstädt-Klein S., Kobiella A., Wolfensteller U., Smolka M.N. (2012). The personality trait self-directedness predicts the amygdala’s reaction to appetizing cues in fMRI. Appetite.

[72] Kaye W.H., Fudge J.L., Paulus M.P. (2009). New insights into symptoms and neurocircuit function of anorexia nervosa. Nat. Rev. Neurosci..

[73] Kaye W.H., Wierenga C.E., Bailer U.F., Simmons A.N., Bischoff-Grethe A. (2013). Nothing tastes as good as skinny feels: The neurobiology of anorexia nervosa. Trends Neurosci..

[74] Jacka F.N., Pasco J.A., Mykletun A., Williams L.J., Hodge A.M., O’Reilly S.L., Nicholson G.C., Kotowicz M.A., Berk M. (2010). Association of Western and traditional diets with depression and anxiety in women. Am. J. Psychiatry.

[75] Lassale C., Batty G.D., Baghdadli A., Jacka F., Sánchez-Villegas A., Kivimäki M., Akbaraly T. (2019). Healthy and unhealthy dietary patterns and risk of depression: A meta-analysis. Mol. Psychiatry.

